# Metabolic barriers in non-small cell lung cancer with *LKB1* and/or *KEAP1* mutations for immunotherapeutic strategies

**DOI:** 10.3389/fonc.2023.1249237

**Published:** 2023-08-22

**Authors:** Ichidai Tanaka, Junji Koyama, Hideyuki Itoigawa, Shunsaku Hayai, Masahiro Morise

**Affiliations:** Department of Respiratory Medicine, Nagoya University Graduate School of Medicine, Nagoya, Japan

**Keywords:** immune checkpoint blockade, NSCLC, LKB1, KEAP1, metabolic barriers, glycolysis, glutaminolysis, PD-1/PD-L1 inhibitors

## Abstract

Currently, immune checkpoint inhibitors (ICIs) are widely considered the standard initial treatment for advanced non-small cell lung cancer (NSCLC) when there are no targetable driver oncogenic alternations. NSCLC tumors that have two alterations in tumor suppressor genes, such as liver kinase B1 (LKB1) and/or Kelch-like ECH-associated protein 1 (KEAP1), have been found to exhibit reduced responsiveness to these therapeutic strategies, as revealed by multiomics analyses identifying immunosuppressed phenotypes. Recent advancements in various biological approaches have gradually unveiled the molecular mechanisms underlying intrinsic reprogrammed metabolism in tumor cells, which contribute to the evasion of immune responses by the tumor. Notably, metabolic alterations in glycolysis and glutaminolysis have a significant impact on tumor aggressiveness and the remodeling of the tumor microenvironment. Since glucose and glutamine are essential for the proliferation and activation of effector T cells, heightened consumption of these nutrients by tumor cells results in immunosuppression and resistance to ICI therapies. This review provides a comprehensive summary of the clinical efficacies of current therapeutic strategies against NSCLC harboring *LKB1* and/or *KEAP1* mutations, along with the metabolic alterations in glycolysis and glutaminolysis observed in these cancer cells. Furthermore, ongoing trials targeting these metabolic alterations are discussed as potential approaches to overcome the extremely poor prognosis associated with this type of cancer.

## 1 Introduction

The advent of immune checkpoint inhibitor (ICI) therapy has revolutionized the treatment approach for various cancers, including advanced non-small cell lung cancer (NSCLC). Currently, the standard first-line therapy for advanced NSCLC without targetable driver oncogenic alternations consists of multiple treatment regimens involving ICIs, either alone or in combination with platinum-based chemotherapy (1–8). Predictors such as programmed death 1 ligand-1 (PD-L1) tumor proportion scores (TPS) or tumor mutational burden (TMB) are available but insufficient in accurately forecasting the treatment outcome (9–11). In first-line therapies for advanced NSCLC, ICIs as monotherapy, such as pembrolizumab and atezolizumab, have demonstrated clinical benefits primarily in patients with high tumor PD-L1 expression (2, 3). However, several combinations of ICIs and platinum-based chemotherapies have been approved as standard first-line therapies irrespective of TPS, although the effectiveness of these combinations still relies to some extent on the tumor PD-L1 expression status. Nonetheless, even among the subset of patients with high tumor PD-L1 expression, approximately 20–30% initially exhibit resistance to ICIs, either alone or in conjunction with platinum-based chemotherapy (1, 9).

Recent multiomics analyses, including next-generation sequencing-based tests (NGS), have played a crucial role in identifying predictive biomarkers for ICI therapies and uncovering mechanisms of immune evasion in cancer (9, 12–15). Among them, T cell–inflamed gene expression profile and proteogenomic characterization in addition to NGS data analysis have revealed that specific driver mutations in NSCLC exhibit discrete immune phenotypes (16, 17). Notably, two tumor suppressor genes, liver kinase B1 (LKB1) and Kelch-like ECH-associated protein 1 (KEAP1), are associated with inactivating driver mutations that contribute to an immunosuppressed phenotype (18). Somatic mutations in *LKB1*, encoded by *serine/threonine kinase 11* (*STK11*), occur in approximately 20-25% of lung adenocarcinoma (LUAD), while inactivating mutations in *KEAP1* are observed in approximately 10-15% of LUAD (19–21). Several studies using a large number of clinical specimens have also reported a high frequency of co-occurring mutations in *STK11* and *KEAP1* (22–24). NSCLC with *STK11* and/or *KEAP1* mutations represents one of most aggressive types of cancer, characterized by resistance to standard cytotoxic chemotherapy or radiotherapy (20, 25–27). However, these tumors also exhibit reduced efficacy to immunotherapy, independent of PD-L1 expression and high TMB (18, 28, 29). This highlights the urgent need for novel therapeutic strategies to effectively treat NSCLC patients with these specific mutations. T-cell infiltration in tumors is known to be relatively weak, and researchers have investigated various factors that contribute to this, such as the secretion of immunosuppressive molecules and impairment of antigen presentation (17, 18). Among these factors, the metabolic reprogramming of glycolysis and glutaminolysis in tumor cells has emerged as a current area of focus for explaining the limited response to immunotherapy (30, 31). The intrinsic metabolic reprogramming of tumor cells, which is essential for tumor growth, also impacts various cells within the tumor microenvironment (TME), leading to immune evasion by the tumor (30–34).

To understand how the inactivation of the two tumor suppressors leads to metabolic reprogramming of glycolysis and glutaminolysis, researchers have gradually uncovered the molecular mechanisms through various biological approaches. These metabolic alterations play a significant role in promoting tumor aggressiveness and reconstructing the TME to support tumor growth (31, 35, 36). In this review, we provide a summary of the current therapeutic strategies and their clinical efficacies against NSCLC with LKB1 and/or KEAP1 inactivation. Furthermore, we delve into the metabolic alterations of glycolysis and glutaminolysis in NSCLC with these mutations, which are associated with ICI resistance, and discuss ongoing trials that target metabolic alterations.

## 2 Clinical efficacies of ICI regimen to advanced NSCLC

### 2.1 Heterogeneity of PD-L1 expression and ICIs efficacy in NSCLC

**Figure 1:**
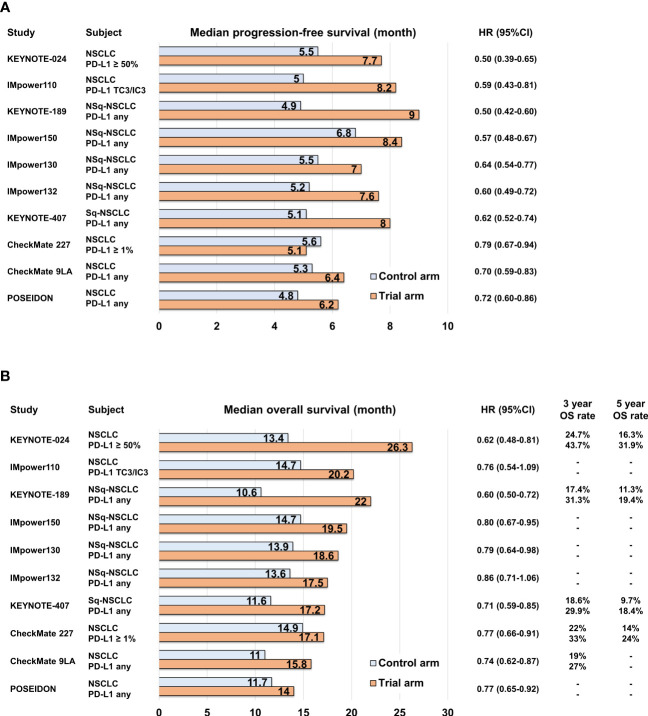
Bar graph comparing control and trial arms in pivotal phase III clinical trials in terms of median progression-free survival **(A)** and median overall survival **(B)**.

PD-L1 expression on cancer cells is regulated by various mechanisms, including inflammatory cytokines, mechanical signals, and tumor-intrinsic cell signaling. Consequently, there is heterogeneity in the PD-L1 expression levels across tumors (37–40), making them imperfect markers for predicting the response to ICIs. However, during the clinical development of anti-PD-1/PD-L1 antibodies, tumor PD-L1 expression status was used for patient selection based on the observed association between the objective response rate of anti-PD-1 antibody, pembrolizumab, and tumor PD-L1 expression level (41). The KEYNOTE-010 study demonstrated the durable response of pembrolizumab in patients with high tumor PD-L1 expression, leading to subsequent KEYNOTE-024 trial that compared pembrolizumab monotherapy with platinum-based chemotherapy specifically in patients with high tumor PD-L1 expression (2, 42). In these trials, which selected patients based on tumor PD-L1 expression status, the anti-PD-1 antibody showed superior survival outcomes compared to platinum-based chemotherapy, and subsequently, the anti-PD-L1 antibody atezolizumab also demonstrated overall superiority over platinum-based chemotherapy (3, 43) (**Figures 1A, B**; **Supplementary Table 1**). Several phase III studies have investigated the clinical efficacy of combining anti-PD-1/PD-L1 antibodies with platinum-based chemotherapy, irrespective of tumor PD-L1 expression, in comparison to platinum-based cytotoxic chemotherapy. These studies, namely, KEYNOTE-189, IMpower150, IMpower130, IMpower132, and KEYNOTE-407, have now become standard first-line options (4, 5, 44–49) (**Figures 1A, B**; **Supplementary Table 1**). In addition, the combination of anti-PD-1 antibody and anti-cytotoxic T-lymphocyte-associated protein 4 (CTLA-4) antibody has also demonstrated similar survival superiority. CheckMate 227 and CheckMate 9LA trials showed that the clinical benefits of nivolumab plus ipilimumab and nivolumab plus ipilimumab in combination with platinum-based chemotherapy, respectively, surpassed those of platinum-based chemotherapy alone (7, 50–52). Moreover, in the phase III POSEIDON study, the combination of anti-PD-L1 antibody durvalumab and anti-CTLA-4 antibody tremelimumab, along with platinum-based chemotherapy, recently showed positive results in terms of both progression-free survival (PFS) and overall survival (OS) when compared to platinum-based chemotherapy alone (8) (**Figures 1A, B**; **Supplementary Table 1**). These combination regimens involving ICIs have emerged as the leading options for standard first-line therapy in advanced NSCLC cases without targetable drive alterations, regardless of TPS and TMB.

In contrast, most molecular-targeted therapies have become established as the standard first-line treatment for NSCLC cases with *epidermal growth factor receptor (EGFR)*, *anaplastic lymphoma kinase (ALK)*, *ROS proto-oncogene 1 (ROS1)*, *B-Raf proto-oncogene (BRAF)*, and *Ret proto-oncogene (RET)* alterations, exhibiting over 50% antitumor response rates and long-term PFS (53). A recent significant advancement in molecular-targeted therapy is the approval of sotorasib for second-line treatment in NSCLC cases with *Kirsten rat sarcoma viral oncogene homolog* (*KRAS*) *G12C* mutation, following immunotherapy-based therapies (54). Interestingly, the presence of oncogenic driver gene mutations has been found to have an impact on the efficacy of ICIs in NSCLC. Specifically, *EGFR* and *KRAS* mutations have been identified as key factors associated with ICI efficacy. NSCLC patients with *KRAS* mutations have shown favorable responses to ICIs with or without platinum-based chemotherapy compared to those without *KRAS* mutations. For instance, in a study involving patients with non-squamous NSCLC treated with pembrolizumab alone or in combination with chemotherapy, those with *KRAS* mutations had a longer PFS compared to patients with wild-type *KRAS* (median PFS 16.5 months vs. 8.0 months) (55). Another study also reported that *KRAS* mutations were significant favorable prognostic factors in NSCLC patients treated with pembrolizumab in combination with carboplatin plus pemetrexed for non-squamous NSCLC or paclitaxel for squamous NSCLC (56). A subgroup analysis of the IMpower150 trial revealed that the combination of atezolizumab, bevacizumab, carboplatin, and paclitaxel (ABCP) showed a greater PFS benefit in the population with *KRAS* mutations compared to the combination of bevacizumab, carboplatin, and paclitaxel, with hazard ratios (HRs) of 0.42 and 0.65, respectively, in *KRAS* mutation-positive and *KRAS* mutation wild-type populations (57).

Conversely, a meta-analysis of phase III studies comparing ICI monotherapy to docetaxel in the pretreatment setting revealed that ICI monotherapy is less beneficial in NSCLC patients with *EGFR* mutant compared to those of wild-type (58). However, several clinical trials have shown the clinical benefit of combining ICIs with platinum-based chemotherapy and an anti-vascular endothelial growth factor (VEGF) strategy. In a subset analysis of the IMpower150 trial, the combination of atezolizumab, carboplatin, paclitaxel, and bevacizumab demonstrated longer PFS and OS compared to carboplatin, paclitaxel, and bevacizumab in patients with common *EGFR* mutations (59). VEGF-A has been found to have an immunosuppressive role by promoting the function of regulatory T-cell and driving the growth of *EGFR* mutant NSCLC. Therefore, combining ICIs with VEGF-A inhibitors, such as bevacizumab, has emerged as an appealing treatment strategy for *EGFR* mutant NSCLC after driver-targeted therapy failure (60–62). However, regarding the predictive value of driver oncogenes other than *EGFR* and *KRAS* mutations, conclusive evidence has not been established at this stage. Several small retrospective cohort studies have reported the efficacy of ICI monotherapy in NSCLC patients with other diver oncogenic alterations, with response rates ranging from 0% in NSCLC patients with *ALK* fusion to 24% in NSCLC patients with *BRAF* mutation (40, 63, 64). Nevertheless, these findings are insufficient to draw definitive conclusions regarding the clinical relevance of ICIs for patients with these driver gene alternations other than *EGFR* and *KRAS*. Regarding *RET* alternations, the ongoing phase III trials comparing the RET inhibitor selpercatinib to other treatments will provide insights into the clinical efficacy of combination therapy involving ICIs for those patients (65).

### 2.2 Therapeutic efficacies of ICI regimens to advanced NSCLC with LKB1 and/or KEAP1 inactivation

**Figure 2:**
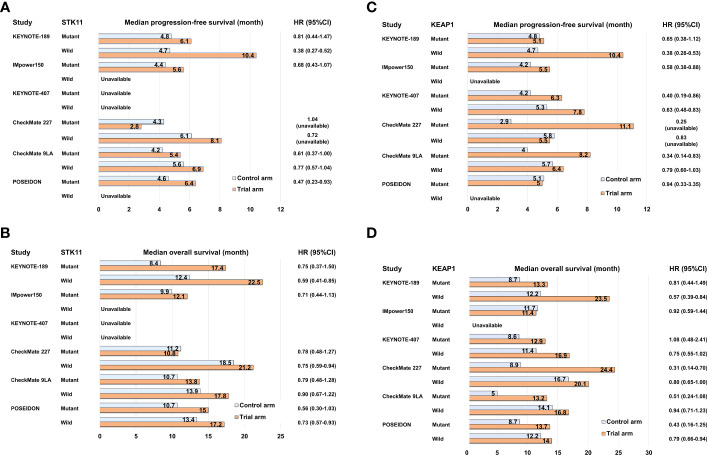
Bar graph comparing control and trial arms in subgroup analyses of pivotal clinical trials for NSCLC with *STK11* or *KEAP1* mutation in terms of median progression-free survival **(A, C)** and median overall survival **(B, D)**.

Recent large-scale profiling studies using NGS in NSCLC have uncovered multiple non-random patterns of driver gene alterations. These patterns often exhibit co-occurrence or mutual exclusivity and are associated with specific driver alterations. One notable example is the co-occurrence of oncogenic driver alterations, such as *KRAS* and *EGFR* mutations, along with the inactivation of well-known tumor suppressor genes like *tumor protein p53* (*TP53)*, *LKB1* (*STK11)*, and *KEAP1*. These co-occurring patterns have significant biological implications and can influence tumor evolution and progression (18). Furthermore, these co-occurring patterns also impact the clinical efficacies of various therapies, including ICI and cytotoxic chemotherapy. In patients with *KRAS*-mutant NSCLC who were treated with ICI monotherapies or ICI combination therapies, the response rate was remarkably higher in the group with *TP53* co-mutation compared to the group with *STK11* co-mutation (28). The median PFS and median OS were reported as 3.0 months and 16.0 months, respectively, for patients with *KRAS*/*TP53* co-mutation (KP group), while it was 1.8 months and 6.4 months for patients with *KRAS*/*STK11* co-mutation (KL group). The underlying biological mechanism explaining the poor efficacy of ICIs in the KL group may be attributed to the immunosuppressive TME caused by LKB1 inactivation followed by *STK11* mutation (18). LKB1 inactivation in cancer cells leads to the production of several immunosuppressive cytokines, such as Interleukin (IL)-6, IL-33, chemokine (C-X-C motif) ligand 7, and granulocyte colony-stimulating factor, which contribute to the mobilization of neutrophils (66). Neutrophils play a role in impeding T-cell movement and function, leading to the development of an “immune desert environment” characterized by reduced tumor-infiltrating lymphocytes. The limited efficacy of ICI monotherapies and ICI combined with cytotoxic chemotherapies has been observed in NSCLC patients with *STK11* or *KEAP1* mutations. In the subgroup analysis of the IMpower150 trial, the *KRAS*-mutant NSCLC patients and co-occurring *STK11* and/or *KEAP1* mutations exhibited a significantly shorter median PFS of the combination therapy ABCP compared to those with wild type in both *STK11* and *KEAP1* (6.0 months vs. 15.2 months) (57) (**Supplementary Table 2**). In contrast, NSCLC patients with *KRAS*/*TP53* co-mutation had a longer median PFS with ABCP compared to those with *KRAS* mutations and wild-type *TP53* (14.3 months vs. 7.3 months) (57). In the subgroup analysis of the KEYNOTE-189 trial, the overall response rate (ORR) of pembrolizumab in combination with platinum plus pemetrexed was 30.6% in NSCLC patients with *STK11* mutation, whereas it was 48.8% in those with *STK11* wild type (67) (**Supplementary Table 2**). Furthermore, in NSCLC patients with *KEAP1* mutation, the ORR of pembrolizumab in combination with platinum plus pemetrexed was 35.6% (67). The median PFS for patients with *STK11* mutation and those with *KEAP1* mutation were 6.1 and 5.1 months (**Figures 2A, C**), respectively, indicating that the clinical efficacy of ICIs combined with cytotoxic chemotherapy is also limited in NSCLC patients with both gene mutations. However, since *STK11* and/or *KEAP1* mutations are also associated with poor clinical outcomes to cytotoxic chemotherapy without ICIs, there may still a benefit in adding PD-1/PD-L1 inhibitors to platinum-based chemotherapy even in this population.

To enhance the clinical outcomes of PD-1/PD-L1 inhibitor-based therapy for “immune desert environment” NSCLC caused by *STK11* and/or *KEAP1* mutations, the addition of CTLA-4 inhibitors to PD-1/PD-L1 inhibitors represents a promising approach. CTLA-4 is expressed on activated T cells upon tumor antigen presentation by dendritic cells. It has a stronger binding affinity to CD80/86 compared to CD28, which is responsible for T-cell activation, thereby suppressing T-cell activation (68). Anti-CTLA-4 antibodies, such as ipilimumab and tremelimumab, block the binding of CTLA-4 to CD80/86, leading to enhanced and sustained T-cell activation (68). The reported clinical benefits of combining PD-1/PD-L1 inhibitors with CTLA-4 inhibitors for NSCLC with *STK11* and/or *KEAP1* mutations are based on exploratory analyses of phase III clinical trials and involve unstratified univariate analysis with a relatively smaller sample size. In the subgroup analysis of CheckMate 227, the PFS-HR of nivolumab plus ipilimumab compared to platinum-base chemotherapy were 1.04 for patients with *STK11* mutation (n = 78) and 0.25 for those with *KEAP1* mutation (n = 38) (69) (**Figures 2A, C**; **Supplementary Table 2**). In the subgroup analysis of CheckMate 9LA, the PFS-HRs of nivolumab plus ipilimumab with platinum-based chemotherapy compared to platinum-based chemotherapy alone were 0.61 (95%CI: 0.37–1.00) for patients with *STK11* mutation and 0.34 (95%CI: 0.14–0.83) for patients with *KEAP1* mutation (52) (**Figures 2A, C**; **Supplementary Table 2**). Further, in the subgroup analysis of the POSEIDON trial, the PFS-HRs of durvalumab plus tremelimumab with platinum-based chemotherapy compared to platinum-based chemotherapy alone were 0.47 (95%CI: 0.23–0.93) for patients with *STK11* mutation (n = 53) and 0.94 (95%CI: 0.33–3.35) for patients with *KEAP1* mutation (n = 28) (70) (**Figures 2A, C**; **Supplementary Table 2**). The subgroup analyses of these major clinical trials indicate that certain ICI combination therapies may have some degree of effectiveness in treating NSCLC with *STK11* or *KEAP1* mutations, although their therapeutic benefits are generally limited (**Figures 2A–D**). Specifically, *KRAS*-mutant NSCLC with *STK11* or *KEAP1* mutations tends to have a poorer prognosis, and comprehensive co-mutation analyses in *KRAS*-mutant NSCLC have not been conducted extensively for other ICI combination therapies except IMpower150 (57). Therefore, for NSCLC cases with these mutations, it is important to continue clinical and molecular analyses and to develop more advanced therapeutic strategies targeting novel therapeutic targets.

## 3 Glycolysis and glutaminolysis in NSCLC with LKB1 and/or KEAP1 inactivation

### 3.1 Glycolysis in NSCLC with LKB1 inactivation

Cancer cells have possess a distinct metabolic characteristic known as the Warburg effect, wherein they preferentially utilize the glycolytic pathway for energy production, even in the presence of sufficient oxygen (71–73). This unique glycometabolism trait is characterized by increased glucose uptake and enhanced carbohydrate conversion into lactose. By consuming high amounts of glucose, tumor cells can rapidly proliferate and generate ATP, while also obtaining the necessary glycometabolic intermediates for synthesizing cellular components (73–75). Glucose is not only vital for tumor cell growth but also plays a crucial role in the proliferation and activation of effector T cells. Consequently, intratumoral effector T cells must outcompete tumor cells to acquire glucose (33, 76). Hence, in rapidly growing tumors, high glucose consumption itself may contribute to immunosuppression. In support of this, a study by Zappasodi et al. explored the correlation between tumor immune infiltration and glycolysis of cancer cells in advanced melanoma patients treated with ipilimumab. They discovered that high expression of glucose catabolism genes in melanoma was inversely associated with infiltration of substantial immune cells, suggesting that tumors with low glycolytic activity are more likely to respond to anti-CTLA-4 antibodies (77). Furthermore, lactate dehydrogenase A (LDHA) and monocarboxylate transporter 1 (MCT1), which are key enzymes involved in glycolysis and lactate production, have been found to exhibit an inverse correlation with immune infiltrates even after ipilimumab treatment (77). This suggests that anti-CTLA-4 blockade alone may be insufficient to enhance immune cell infiltration in highly glycolytic tumors.

**Figure 3:**
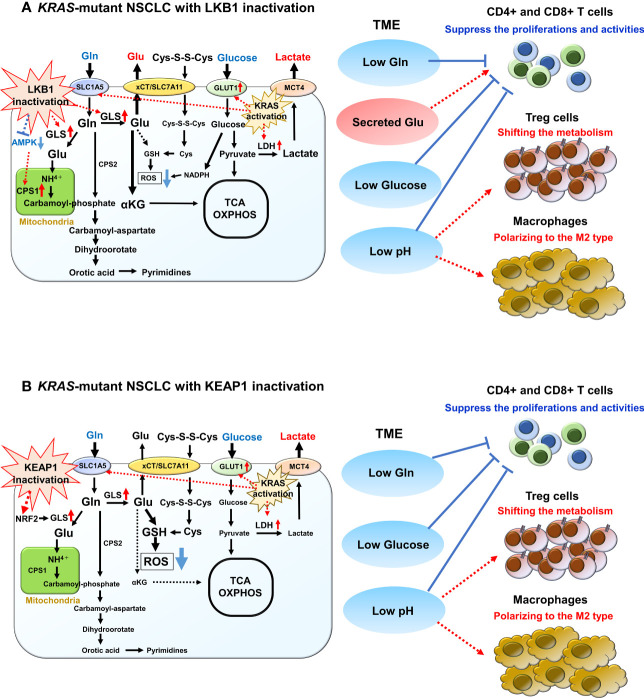
Overview of glucose and glutamine (Gln) metabolism in *KRAS*-mutant NSCLC with LKB1 or KEAP1 inactivation. **(A)** Overview of glucose and Gln metabolism in *KRAS*-mutant NSCLC with LKB1 inactivation. Glucose is imported by glucose transporter 1 (GLUT1) and is then metabolized by glycolysis into pyruvate. Pyruvate then either enters the tricarboxylic acid (TCA) cycle for ATP synthesis or is converted to lactate by lactate dehydrogenase (LDH). Mutant-*KRAS* enhances aerobic glycolysis by upregulating GLUT1 and LDH. After exported by monocarboxylate transporter 4 (MCT4), lactate increases extracellular acidification rate of tumor microenvironment (TME) and has diverse effects on various immune cells. Gln is imported by SLC1A5 where it then enters into the mitochondria and is converted to glutamate (Glu) by glutaminase (GLS), which is highly increased in NSCLC with LKB1 inactivation. The released NH4+ during the conversion to Glu is used for the synthesis of the purine/pyrimidine base. Carbamoyl phosphate synthetase 1 (CPS1), which is the first rate-limiting mitochondrial enzyme in the urea cycle, is overexpressed in NSCLC with LKB1 inactivation. Glu is also used for the precursor of glutathione (GSH), which promotes reactive oxygen species (ROS) detoxification. In addition, the excess synthesized Glu is excreted out via xCT/SLC7A11 and is then required for T-cell activation. **(B)** Overview of glucose and Gln metabolism in *KRAS*-mutant NSCLC with KEAP1 inactivation. Glucose and Gln metabolism are promoted to the anabolic pathway through interaction with the phosphatidylinositol 3’-kinase/protein kinase B signaling. Gln exchanges Glu for cystine via the antiporter xCT (SLC3A2/SLC7A11), which activated NRF2 target. Both cystine and Gln are used to produce GSH, leading to ROS neutralization. Gln is also used in purine base synthesis, and fewer Gln are used for TCA cycle.

LKB1 is recognized as a key metabolic regulator that exerts control over glucose metabolism by inducing the expression of critical genes encoding enzymes involved in glycolysis, gluconeogenesis, aerobic oxidation, and the pentose phosphate pathway. It achieves this regulation by acting on several downstream targets, including the central metabolic sensor called AMP-activated protein kinase (AMPK) (78–82). Under conditions of energy stress, LKB1 directly phosphorylates AMPK, which in turn promotes the activation of catabolic pathways such as glycolysis and fatty acid oxidation. Simultaneously, it suppresses anabolic pathways, including gluconeogenic enzymes, to maintain intracellular ATP levels (81, 82). Furthermore, the LKB1-AMPK axis plays a role in regulating cell growth and division by inhibiting the mammalian target of rapamycin complex 1 (mTORC1), which serves as the central integrator of nutrient and mitogenic signals. Notably, mTORC1 is often activated in cancer cells, contributing to tumor progression (81, 83). When LKB1 function is compromised, these downstream factors become dysregulated, leading to increased glucose uptake and consumption, as well as a metabolic shift toward aerobic glycolysis. Even in benign tumors with LKB1 haploinsufficient, there have been reports of enhanced accumulation of 18F-deoxyglucose on positron emission tomography, indicating that the loss of LKB1 function directly influences glucose metabolic reprogramming (84). Studies using the naturally LKB1-inactivated NSCLC cell line A549 have demonstrated that the activation of hypoxia-inducible factor 1 alpha (HIF-1α), induced by LKB1 inactivation, contributes to the enhancement of the aerobic glycolytic system (85). The absence of LKB1 was found to result in increased HIF-1α expression, which was shown to depend on both mTOR signaling and cellular mitochondrial reactive oxygen species (ROS) levels. Notably, *HIF-1α* knockdown in LKB1-deficient cell line significantly reduced proliferation under low-glucose conditions, indicating that HIF-1α promotes the growth of NSCLC with LKB1 inactivation even when nutrients are limited (85). Alongside LKB1 inactivation, *KRAS* mutation, which is the most prevalent oncogenic alteration in tumors with LKB1 inactivation, also leads to heightened glucose uptake and increased glycolytic activity. This is achieved through the upregulation of glucose transporter 1 (GLUT1) and key glycolytic enzymes such as LDHA, hexokinases, and phosphofructokinase 1 (PFK1) (86–88). Mutant *KRAS*, by upregulating GLUT1 and these glycolytic enzymes, further enhances aerobic glycolysis. Therefore, lung cancer cells with simultaneous LKB1 inactivation and *KRAS* mutation are likely to exhibit greater glucose uptake and consumption, contributing to their rapid tumor growth and suppression of intratumor effector T-cell activity (**Figure 3A**).

Tumor cells that undergo rapid proliferation stimulate the formation of tumor blood vessels by releasing factors that promote angiogenesis. This process is crucial for acquiring nutrients and oxygen. However, the resulting vasculature is often immature and hyperpermeable, leading to the development of hypoxic regions within the tumor. These hypoxic areas create a barrier that hampers the infiltration of immune cells (89). Moreover, the hypoxic tumor microenvironment contributes to the accumulation of immunosuppressive metabolic byproducts. These metabolic alterations negatively impact the function of effector T cells, while they may have little to no effect or even benefit suppressive immune populations like regulatory T cells (Treg) and suppressive myeloid populations (31, 90). The increased glycolytic activity in tumor leads to the production of large amounts of lactate, which in turn acidifies the extracellular spaces. NSCLC with LKB1 inactivation is associated with an elevated extracellular acidification rate (ECAR), which indicates higher lactate levels. Introducing transient expression of LKB1 in an NSCLC cell line with LKB1 inactivation resulted in a 20% decrease in ECAR (85). Despite its ability to lower pH, lactate has diverse effects on immune cell populations. For instance, it promotes a metabolic shift in Treg to enable their activity in low-glucose environments and induces macrophages to adopt an M2 phenotype, which supports tumor growth (91–93). Notably, the accumulation of lactic acid can suppress the proliferation of CD4+ and CD8+ T cells, as well as inhibit their cytokine production (94). Lactate can deplete intracellular nicotinamide adenine dinucleotide+ (NAD+) levels and impair effector T cells because LDH uses lactate to generate NAD+ hydrogen (NADH). Conversely, Treg can continue to function in high lactate environments where conventional T cells are suppressed due to the NAD+ produced by mitochondrial metabolism (91, 93).

Furthermore, it is recognized that circulating lactate is transported into cells via MCT1 and used as an energy source and substrate for lipogenesis in certain cancer types (95). In an analysis that measured the uptake of metabolic intermediates from tumor samples after labeled glucose infusion in NSCLC patients, elevated lactate labeling was observed, indicating the uptake of lactate in tumors compared to glycolytic metabolites (96). In addition, a xenograft model using an NSCLC cell line with LKB1 inactivation showed increased labeled lactate in the tumor, indicating the uptake of extracellular lactate and its incorporation into the tricarboxylic acid (TCA) cycle as a carbon source (96). This study suggests that lactate plays a crucial role as an energy source in LKB1-inactivated NSCLC. Apart from LDHA/B, elevated levels of the lactate transporter MCT1/4 have also been observed in lung cancer cells with LKB1 inactivation (96, 97), suggesting that intracellular lactate is not only incorporated into the TCA cycle but that extracellular lactate released by neighboring cancer cells can be taken up and incorporated into the TCA cycle as an energy source (**Figure 3A**).

### 3.2 Glutaminolysis in LKB1-inactivated NSCLC

Glutamine (Gln) is a vital amino acid with significant roles in cellular functions, including energy and biomolecule synthesis, as well as ROS scavenging. Upon cellular uptake, Gln is converted into glutamate (Glu) by the enzyme glutaminase (GLS). It is further converted to α-ketoglutarate, which enters the TCA cycle, generating metabolic intermediates for lipid, nucleic acid, and protein synthesis. In the TME, both tumor cells and infiltrating immune cells have a high demand for Gln, similar to glucose. T-cell activation and proliferation heavily rely on Gln metabolism, and when Gln is insufficient in the TME, the high consumption by tumors can inhibit T-cell activity. Conversely, reduced Gln metabolism in tumors has shown to increase Gln utilization within the TME (34). In a mouse model of colorectal cancer using MC38 tumor-bearing mice, combination therapy of anti-PD-1 monoclonal antibody and a Gln antagonist prodrug, 6-diazo-5-oxo-L-norleucine, resulted in enhanced tumor growth inhibition (98).

Several oncogenes and tumor suppressors play a role in regulating Gln metabolism, and LKB1 inactivation is also implicated in Gln flux regulation. LKB1 ectopic expression in NSCLC cells with LKB1 deficiency led to a decrease in Gln-derived Glu (85). Furthermore, in the LKB1-deficient NSCLC cell line A549, the majority of Gln-derived carbon entered the TCA cycle compared to glucose-derived carbon, in contrast to the cell line with LKB1 ectopic expression (85). Moreover, LKB1-inactivated NSCLC cells exhibit higher levels of GLS expression and more active conversion of Gln to Glu (99). The released NH4+ during this conversion is used for the synthesis of purine/pyrimidine bases, which are essential for rapid cell proliferation. Notably, studies have demonstrated characteristic overexpression of carbamoyl phosphate synthetase 1 (CPS1), the first rate-limiting mitochondrial enzyme in the urea cycle, in a subset of NSCLC with LKB1 inactivation (100, 101). CPS1 plays a vital role in promoting cell growth by increasing the bioavailability of carbamoyl phosphate, an intermediary metabolite required for *de novo* pyrimidine synthesis. The CPS1 expression is transcriptionally regulated by LKB1 through AMPK, and cases with high CPS1 expression have been associated with poor prognosis, particularly in NSCLC with LKB1 inactivation (100, 101). Thus, LKB1-inactivated lung cancers effectively utilize excess Gln, and the activation of these metabolic pathways may contribute to their high malignancy. Furthermore, oncogenic KRAS has been shown to stimulate Gln catabolism in the mitochondria (87, 88). Since both KRAS and LKB1 regulate metabolism, the co-mutation of these two genes could lead to a unique metabolic phenotype not observed with either mutation alone. In fact, CPS1 plays a pivotal role in maintaining the balance between purine and pyrimidine in NSCLC cells with co-mutated *KRAS* and *LKB1*, and the enzyme also provides an alternative pool of carbamoyl phosphate to sustain pyrimidine availability (101). Hence, apart from glucose metabolism, reprogramming of Gln metabolism in tumors harboring co-mutated *KRAS* and *LKB1* likely contributes to aggressive oncological behavior and impacts TME (**Figure 3A**). Notably, the clinical response to PD-(L)1 inhibition is significantly poorer in NSCLC patients with co-mutated *KRAS* and *STK11* compared to those with only *STK11* mutation (102).

Cellular metabolism generates ROS, which need to be scavenged to prevent damage to DNA, RNA, and proteins. Gln metabolism also plays an important role in maintaining oxidative homeostasis. Glu, generated from Gln by the catalytic action of GLS, serves as the precursor of glutathione (GSH), which promotes ROS detoxification (103). GSH, along with thioredoxin, plays a major role in neutralizing ROS and is synthesized through an NADPH-dependent mechanism. Loss of LKB1 activity resulting in metabolic reprogramming leads to elevated ROS levels and metabolic stress, while the conversion of Gln to Glu significantly contributes to ROS neutralization by stimulating the production of GSH (104). Furthermore, due to the increased aerobic glycolysis in cancer cells, metabolites are shunted toward the pentose phosphate pathway (PPP), which aids in ROS scavenging. In LKB1 mutant cell lines, such as A549 and H460 cells, genes associated with the PPP are upregulated, indicating their dependence on this pathway (105). Meanwhile, A549 cells that re-express LKB1 exhibit a higher apoptosis rate under ROS stress compared to control cells (104), suggesting that the upregulation of Gln conversion observed in *LKB1*-inactivating mutations may confer increased resistance to ROS.

### 3.3 Glutaminolysis in KEAP1-inactivated NSCLC

The KEAP1-nuclear factor erythroid-derived 2-like 2 (NRF2) pathway plays a crucial role in regulating the cellular response to oxidative stress, and its signaling abnormalities have been observed in various cancer types, including NSCLC (106, 107). In normal conditions, KEAP1 ubiquitinates NRF2, encoded by the *NFE2L2* gene, for degradation through ubiquitination. However, under stress conditions, KEAP1 activity is reduced, leading to increased transcription of NRF2 target genes. This activation of NRF2 signaling enhances antioxidant defense against ROS and regulates drug detoxification and immune response (106, 107). In NSCLC, *KEAP1* deficiency is commonly observed in LUAD, while activating alterations of *NFE2L2* are more prevalent in squamous cell lung carcinoma (~20%), with both alterations being mutually exclusive (108). The constitutive activation of NRF2 signaling in advanced cancer patients diminishes the therapeutic effects of chemotherapy and radiation therapy, as these treatments rely on inducing cell death through DNA replication damage and ROS induction (106, 107). Furthermore, recent studies have revealed that NRF2 activation promotes various metabolic reprogramming processes and is associated with tumor progression in NSCLC, including glutaminolysis (109–111).

Similar to tumors with LKB1 inactivation, tumors harboring KEAP1 mutations increased uptake of Gln from TME, leading to reduced availability of Gln for infiltrating T cells and consequent inhibition of their activation. Activation of NRF2 signaling resulting from KEAP1 inactivation promotes glucose and Gln metabolism toward the anabolic pathway through phosphatidylinositol 3’-kinase/protein kinase B signaling (112). This increased Gln consumption is accompanied by increased expression of the Gln importer SLC1A5 (113). Furthermore, the incorporated Gln exchanges Glu for cystine through the antiporter xCT (SLC3A2/SLC7A11), which is upregulated as a target of NRF2 activation, in a Gln degradation-dependent manner (113, 114). Both cystine and Gln contribute to the production of GSH, thereby enhancing antioxidant activity. In addition, Gln is actively used in purine base synthesis. Therefore, tumors with KEAP1 inactivation may have limited Gln availability for the TCA cycle (**Figure 3B**). NRF2 knockdown in NSCLC cell lines, such as A549, reduces GSH formation from Gln (104). Furthermore, *KEAP1*-mutant NSCLC cell lines demonstrate sensitivity to GLS inhibition due to their high dependence on Gln uptake in the culture medium (104). Integrating these findings, the combination of GLS inhibition and immunotherapy may offer a promising therapeutic strategy in KEAP1-inactivated NSCLC. By suppressing Gln uptake, this strategy could potentially activate T cells in the TME while attenuating the antioxidant effect of KEAP1-inactivated tumors. Furthermore, Pranavi et al. found that NSCLC with KEAP1 inactivation exhibits increased dependence on glucose under glucose-limiting conditions, as NRF2-dependent SLC7A11 expression is upregulated, resulting in cytotoxicity related to disulfide stress (115). In addition, they demonstrated the high sensitivity of KEAP1-inactivated NSCLC to GLUT inhibitor (115). These findings suggest that targeting Gln and glucose metabolism could be an attractive therapeutic target in NSCLC cases with KEAP1 inactivation or constitutive activation of NRF2.

### 3.4 Glutaminolysis in NSCLC with co-occurring mutations of *STK11* and *KEAP1*

Clinical data analysis reveals that lung cancers characterized by simultaneous mutations in LKB1 and KEAP1 exhibit an exceptionally poor prognosis (23). *In vitro* and *in vivo* studies have demonstrated that co-occurring mutations of *STK11* and *KEAP1* in *KRAS*-mutant NSCLC promote tumor growth and confer enhanced resistance to radiotherapy (116). The co-inactivation of LKB1 and KEAP1 cooperatively promotes metabolic reprogramming in *KRAS*-mutant tumor, and even in the presence of KEAP1 inactivation, LKB1 inactivation modulates NRF2 activity through increased ROS levels (104). *LKB1*-mutant cells induce NRF2-dependent Glu cysteine ligase expression, a key enzyme that generates γ-Gly-Gly from Gln and cysteine to increase the GSH pool (104). These results indicate that *KRAS*-mutant NSCLC with co-inactivation of LKB1 and KEAP1 enhanced Gln dependence compared to *KRAS*-mutant NSCLC with LKB1 or KEAP1 inactivation. Consistently, *KRAS*-mutant NSCLC cell lines with co-inactivation of LKB1 and KEAP1 display increased sensitivity to GLS inhibitors compared to other cell lines (104), indicating that targeting glutaminolysis in KRAS-mutant NSCLC with co-inactivation of LKB1 and KEAP1 holds promise as a therapeutic strategy.

In a study conducted by Best et al., distinct metabolic characteristics were observed among KRAS-KEAP1 (KK), KRAS-LKB1 (KL), and KRAS-KEAP1-LKB1 (KKL) mutant LUAD using genetically engineered mouse models (99). In *KRAS*-mutant LUAD with LKB1 inactivation, the expression of GLS1, an enzyme responsible for metabolizing Gln to Glu, was significantly higher compared to *KRAS*-mutant NSCLC with co-inactivation of LKB1 and KEAP1. The conversion of Gln to Glu was particularly enhanced in the KL mouse model (**Figure 3A**). Furthermore, the influx of α-ketoglutaric acid into the TCA cycle was significantly increased in KL mice compared to KK or KKL mice (99). Tumors from KL mice also exhibited a notable increase in orotic acid, which is synthesized during the Gln to Glu conversion process via carbamoyl phosphate. Orotic acid is a precursor of pyrimidine and its synthesis directly affects pyrimidine production (99). Tumors from KL mice also exhibited a notable increase in orotic acid, which is synthesized during the Gln to Glu conversion process via carbamoyl phosphate. Orotic acid is a precursor of pyrimidine and its synthesis directly affects pyrimidine production (117, 118). Increased orotic acid synthesis is closely linked to enhanced nucleic acid synthesis, as nucleotide synthesis is tightly regulated by pyrimidine. In *KRAS*-mutant LUAD with LKB1, CPS1, an enzyme responsible for carbamoyl phosphate synthesis in the mitochondria, is highly expressed, and the heightened Gln metabolism contributes to rapid tumor growth through increased nucleic acid synthesis (100, 101). Excess Glu synthesized is also released from cancer cells via xCT/SLC7A11 (119, 120). Best et al. demonstrated that the release of Glu from cancer cells is crucial for T-cell activation and clonal expansion of T-cell receptors (99). Therefore, GLS inhibition attenuates CD8+ T-cell activation, suggesting that the combining GLS inhibitors with immunotherapy may not enhance the immune response. Particularly in KL mice, the amount of Glu released from cancer cells was higher, and KKL mice exhibited a similar Glu metabolic pattern to KL mice compared to KK mice (99). These findings suggest that GLS inhibitors may be less effective in *KRAS*-mutant LUAD with LKB1 inactivation and co-occurring mutations of LKB1 and KEAP1 compared to *KRAS*-mutant LUAD with KEAP1 inactivation.

## 4 Discussion

To date, subgroup analyses of pivotal clinical trials have shown that current ICI combination regimens have some effectiveness in NSCLC patients with *LKB1* or *KEAP1* inactivation compared to standard platinum doublet chemotherapies (52, 57, 67, 69, 70). However, their efficacy is not sufficient to significantly improve long-term prognosis compared to NSCLC patients without *LKB1* and *KEAP1* inactivation (52, 57, 67, 69, 70). This indicates that the combination of anti-PD-1/PD-L1 antibodies with cytotoxic chemotherapies and/or anti-CTLA-4 antibodies is unable to fully restore the dysfunctional state of T cells or NK cells in NSCLC with these mutations. Moreover, the clinical efficacy of most regimens has not yet been analyzed for *KRAS*-mutant NSCLC with LKB1 or KEAP1 inactivation, which is associated with the poorest prognosis (29, 102). On the other hand, a subgroup analysis of the IMpower150 trial revealed that the trial arm, ABCP regimen, demonstrated superior antitumor effects compared to the control arm in *KRAS*-mutant NSCLC with *STK11* or *KEAP1* mutations (57). By normalizing abnormal tumor vasculature, the addition of VEGF-A inhibitors to ICIs can increase the infiltration of effector T cells into tumors (121). Furthermore, since VEGF-A receptors are expressed on various tumor-promoting immune cells, such as Tregs and immature dendritic cells, this combination therapy may have additional effects in converting the intrinsically immunosuppressive TME into an immunosupportive one, even in immune cold subtypes (121). However, further analysis is needed to fully understand the significance of VEGF-A inhibition for immune cold tumors from both basic and clinical perspectives. Regarding molecular-targeted agents for *KRAS G12C* mutations, sotorasib and adagrasib are now indicated as a second-line treatment following ICI regimens and has expanded the therapeutic options for *KRAS*-mutant NSCLC patients (54). However, its efficacy is limited in cases of NSCLC with co-mutations of *STK11* and *KEAP1* (122). Similarly, in NSCLC with *EGFR* mutations, co-mutations such as *TP53* and *RB transcriptional corepressor 1* can affect the antitumor effect of EGFR-tyrosine kinase inhibitors (123). Therefore, in addition to targeting oncogenic driver alterations, it is increasingly important to identify inactivating mutations in tumor suppressor genes that can impact the efficacy of immunotherapy and of molecularly targeted agents. In fact, some clinical trials of novel molecularly targeted agents targeting *KRAS G12C* mutation have included *STK11* mutation as a stratification factor (124, 125) (**Table 1**). These trends underscore the need for novel therapeutic strategies in the treatment of NSCLC with *STK11* and/or *KEAP1* mutations, as the efficacy of ICIs and molecular targeting agents directly affects patient outcomes.

**Table 1:** Summary of on-goiong trials against advanced NSCLC with *STK11* or *KEAP1* mutation.

Study	Subject of research	Treatment setting	Treatment regimen	Overcoming mechanism	Phase	Primary outcome
Ongoing trials where *STK11* mutation is a stratification factor						
CodeBreaK201 NCT04933695 (124)	*KRAS G12C* mutant NSCLC with PD-L1 < 1%, stratified by *STK11* co-mutation	Treatment naïve	AMG510 (Sotorasib)	KRAS G12C inhibitor	Phase 2	ORR
KRYSTAL-1 NCT03785249 (125)	Solid tumor harboring *KRAS G12C* mutation, stratified by *STK11* co-mutation	Previously treated	MRTX849 (Adagrasib)	KRAS G12C inhibitor	Phase 1/2	Safety, ORR
Ongoing trials						
FAME NCT03709147 (126)	LUAD with LKB1 inactivation	Treatment naïve	Platinum+PEM+Pembrolizumab+Metfolmin Platinum+PEM+Pembrolizumab+Metfolmin+FMD	Biguanide and Nutrient Deprivation	Randomized Phase 2	PFS
BeGIN NCT03872427 (127)	Solid tumor harboring *NF1/KEAP1/STK11* mutation	Previously treated	CB-839 (Telaglenastat)	Glutaminase inhibitor	Phase 2	ORR
NCT04471415 (128)	NSCLC harboring *KEAP1/NFE2L2/STK11* alteration	Previously treated	DRP-104 (Sirpiglenastat)	Glutamine antagonist	Phase 1/2a	Safety, ORR
CAPTUR NCT03297606 (129)	Solid tumor harboring *STK11/NF1/NF2/*other mutation	Previously treated	Temsirolimus	mTORC1 inhibitor	Phase 2	ORR
NCT05469178 (130)	NSq-NSCLC harboring *STK11* mutation	Treatment naïve	CBDCA+PEM+Pembrolizumab+Bemcentinib	AXL inhibitor	Phase 1b/2a	DLT, ORR
NCT05704634 (131)	NSCLC harboring *STK11* mutation	Previously treated	Cemiplimab+Sarilumab	IL6-receptor antibody	Phase 1b	Safety, ORR
NCT05275868 (132)	NSCLC harboring *NFE2L2/KEAP1/CUL3* alteration	Previously treated	MGY825	unavailable	Phase 1	Safety
KontRASt-06 NCT05445843 (133)	*KRAS G12C* mutant NSCLC harboring co-mutation of *STK11* and PD-L1 ≥ 1%	Treatment naïve	JDQ443 (Opnurasib)	KRAS G12C inhibitor	Phase 2	ORR
NCT05276726 (134)	NSCLC harboring co-mutation of *KRAS G12C* and *STK11* and *KEAP1* wild-type	Any	JAB-21822	KRAS G12C inhibitor	Phase 1b/2	DLT, ORR

NSCLC, Non-small cell lung cancer; NSq-NSCLC, Non-squamous non-small cell lung cance; LUAD, Lung adenocarcinoma; LKB1, Liver kinase B1; STK11, Serine/threonine kinase 11; KEAP1, Kelch-like ECH-associated protein 1; NEF2L2, Nuclear factor erythroid 2-related factor 2; CUL3, Cullin3; KRAS, Kirsten rat sarcoma virus; mTORC1, Mammalian target of rapamycin complex 1; AXL, AXL receptor tyrosine kinase; IL-6, Interleukin 6; PFS, Progression-free survival; HR, Hazard ratio; DLT, Dose limiting toxicity; ORR, Overall response rate; CBDCA, Carboplatin; PEM, Pemetrexed.

Concurrent with the advancements in immune checkpoint inhibitors (ICIs) and molecularly targeted therapies, recent fundamental research has uncovered that each driver gene alteration has a cancer-specific impact on the TME through metabolic reprogramming. Specifically, the alteration of glucose and glutamine (Gln) metabolism resulting from LKB1 or KEAP1 inactivation appears to play a significant role in diminishing the effectiveness of current immunotherapies by suppressing the activity of effector T cells. Ongoing clinical trials targeting glucose or Gln metabolism, as depicted in **Table 1**, aim to develop novel therapies for NSCLC with LKB1 or KEAP1 inactivation (126–128).

One therapeutic strategy being explored involves the addition of metformin, a commonly used medication for type 2 diabetes, to cytotoxic chemotherapy. Accumulating evidence supports the antitumor effects of metformin, as it enhances AMPK-mediated cell growth inhibition and cisplatin-induced apoptosis in LKB1-inactivated NSCLC (135, 136). Interestingly, despite initial reports indicating that metformin requires LKB1 for the regulation of gluconeogenesis in the liver, it demonstrates efficacy in LKB1-inactivated NSCLC (137). Clinical trials targeting Gln metabolism have also been initiated, employing Gln antagonists and oral GLS inhibitors, to explore a new therapeutic approach for NSCLC with LKB1 inactivation or KEAP1 inactivation/NFE2L2 alteration (127, 128) (**Table 1**). However, the utilization of glutamate (Glu) released from cancer cells by T cells reveals a complex and interconnected relationship between cancer metabolism and immune cells within the TME (99). Furthermore, NSCLC with concurrent STK11 and KEAP1 mutations exhibit distinct Gln metabolism patterns compared to NSCLC with KEAP1 mutation alone, suggesting that the antitumor effects of targeting Gln metabolism may vary among NSCLC subgroups with different mutation co-occurring patterns (99). Therefore, considering the potential impact of diverse metabolic reprogramming based on specific mutation patterns, it will be crucial to assess the response of each mutated subgroup when treated with Gln metabolism inhibitors, either alone or in combination with a PD-(L)1 inhibitor.

In conclusion, high consumption of glycolysis and glutaminolysis in immune-resistant phenotype tumors, such as NSCLC with LKB1 and/or KEAP1 inactivation, not only contribute to tumor aggressiveness but also impede intratumor T-cell function. The presence of co-occurring mutations in NSCLC leads to distinct metabolic alterations that impact immune cells within TME. These differences in metabolic reprogramming may affect clinical efficacies of current ICI combination regimens and novel agents targeting metabolic enzymes. To develop new therapeutic strategies that target metabolic alterations in combination with ICI regimens for NSCLC with LKB1 and/or KEAP1 inactivation, further extensive analyses on a larger scale will be necessary.

## Author contributions

IT, JK, HI, SH, and MM were involved in the initial drafting of the manuscript, data collection, and analysis. They also contributed to the conceptualization of the study, reviewed the manuscript, and provided feedback and edits. All authors have read and given their approval for the final version of the manuscript. IT have demonstrated the dependency of CPS1, a metabolic enzyme, in cell growth, metabolism and prognosis in LKB1-inactivated lung adenocarcinomas. Furthermore, Serglycin secretion, which is a chondroitin sulfate proteoglycan involved in reprograming to an immunosuppressive TME, is epigenetically induced through nicotinamide N-methyltransferase-induced perturbation of methionine metabolism in TTF-1–negative lung adenocarcinoma. These results were published in J Natl Cancer Inst (2017) 109:1-9 and J Natl Cancer Inst (2022) 114:290-301.
